# Mental Workload in Neuropsychology: An Example With the NASA-TLX in Adults With HIV

**DOI:** 10.3389/fnrgo.2022.881653

**Published:** 2022-05-11

**Authors:** David J. Hardy, Charles H. Hinkin

**Affiliations:** ^1^Department of Psychology, Loyola Marymount University, Los Angeles, CA, United States; ^2^Department of Psychiatry and Biobehavioral Sciences, David Geffen School of Medicine, University of California, Los Angeles, Los Angeles, CA, United States; ^3^VA Greater Los Angeles Healthcare System, Los Angeles, CA, United States

**Keywords:** neuroergonomics, workload, mental workload, neuropsychology, HIV, NASA-TLX, Multi-Attribute Task Battery

## Abstract

A preliminary set of analyses are presented, where workload was examined in 32 adults infected with the human immunodeficiency virus (HIV). Like the current COVID-19 pandemic (caused by the SARS-CoV-2 virus), HIV can produce a wide variety of symptoms, including various levels of cognitive dysfunction. In fact, a recent meta-analysis estimates that of the 39 million adults infected globally with HIV, 42.6% exhibit some form of HIV-associated neurocognitive disorder. A common cognitive symptom in HIV is decline in attention and executive functioning. Though typically examined by clinicians with less precise traditional paper-and-pencil neuropsychological tests, we examined this aspect of cognitive functioning using a more psychometrically sophisticated task as we had HIV-positive adults perform a computerized tracking task in single, dual, and tri-task conditions *via* the Multi-Attribute Task (MAT) Battery. Also assessed was mental workload, with the NASA-Task Load Index (NASA-TLX), rarely used in neuropsychology but a standard tool in human factors and neuroergonomics research. As expected, tracking performance declined with task condition difficulty (*p* < 0.001). Although no direct statistical comparisons were made, MAT performance here appeared worse than the MAT performance of various other groups reported in the research literature and in our laboratory. Ratings of workload also tended to increase as a function of task condition difficulty (*p* < 0.001). Plotting MAT tracking performance against the Mental Demand subscale scores, large individual differences in this aspect of workload were evident in both optimal and sub-optimal tracking performance. To examine likely variables with a potential impact on Mental Demand, a variety of variables (nadir CD4 count, viral load, depression symptoms, diagnosis of AIDS, presence of opportunistic infection, general cognitive status, etc.) were examined in relation to the Mental Demand scale, with age showing a significant association (*r* = 0.41, *p* = 0.022) and a diagnosis of AIDS showing trend associations (*p*s ≥ 0.066). Findings suggesting a deficit in metacognition or insight are also discussed. It is argued that assessment of workload (and its various aspects or components) can provide valuable additional information in neuropsychology.

## Introduction

Like the current COVID-19 pandemic (caused by the SARS-CoV-2 virus), infection with the human immunodeficiency virus (HIV) can produce an array of symptoms including various levels of neurocognitive dysfunction. In fact, a recent meta-analysis estimates that of the 39 million adults infected worldwide with HIV, 42.6% exhibit some form of HIV-associated neurocognitive disorder (HAND; Wang et al., 2020). That such cognitive symptoms persist in a large segment of adults with HIV despite the wide availability and use of anti-retroviral treatments is of continued interest and concern to researchers and clinicians. Most research on the cognitive functioning of this clinical population involves traditional standardized tests of clinical neuropsychology, most of which are so-called paper-and-pencil tests. The strengths of these traditional measures include the standardization of testing procedures and the development of comparative or normative data. With such test norms it is possible to determine degree of cognitive impairment. These tests have also been shown to be sensitive to dysfunction or disruption of nervous system functioning. However, to examine more specific aspects of cognitive processes, a small subset of neuropsychological studies has used more experimentally oriented tasks based on cognitive psychology and cognitive neuroscience methods (e.g., Martin et al., 2018; Hardy et al., 2020; for reviews see Hardy and Hinkin, 2002; Woods et al., 2009).

An experimental task that has never been used in the cognitive assessment of adults with HIV (or as far as we are aware any clinical population in neuropsychology) is the Multi-Attribute Task (MAT) Battery developed by Comstock and Arnegard (1992) (see also Cegarra et al., 2020; https://matb.larc.nasa.gov). The MAT, originating from human factors and neuroergonomics research, was designed as a task simulation of the multiple activities involved with piloting an aircraft. Because a specific and frequent cognitive symptom in HIV is decline in attention and executive functioning (Woods et al., 2009), the multi-tasking aspects of the MAT should make it an ideal assessment tool in the cognitive assessment of adults with HIV. In the present study, we focus on the continuous tracking task of the MAT, where participants will perform the tracking task alone (a single-task condition), while performing one other task (a dual-task condition), and while concurrently performing two other tasks (the tri-task condition). The simulation aspect of the MAT, including the continuous nature of all the sub-tasks (none of these are presented trial-by-trial as in a typical computerized task), also provides a greater sense of ecological validity relative to the static and highly abstract nature of typical clinical and experimental tests of cognitive functioning.

A central focus of the present study is the examination of workload in a clinical population. Workload can be described as the relationship between the processing resources (mental, physical, etc.) available to the individual and the various demands of the task he or she is performing (Hart and Staveland, 1988; Parasuraman et al., 2008). Thus, workload provides a measure of distinction between the state of the operator (the person performing the task) and behavioral performance. As has been recently noted (Hardy and Wright, 2018; Hardy, 2021), the concept and measure of mental workload, or any aspect of workload, is surprisingly uncommon in the field of neuropsychology. It is argued that workload can be a useful adjunct measure in neuropsychological assessment.

Workload was assessed in the present study with the NASA Task Load Index (NASA-TLX; Hart and Staveland, 1988; Hart, 2006; see www.nasatlx.com). This measure includes various subscales (details are provided in the Method section) and it is predicted that reported workload will increase as task demands increase in the single, dual, and tri-task conditions of the MAT Battery. This should be especially evident in the more cognitive-specific subscales, such as Mental Demand, and perhaps less so in a subscale such as Physical Demand. Furthermore, because of normal individual differences and the exacerbated individual differences in cognitive status that often seem to be the case in adults with HIV (and in many clinical syndromes), we expect large individual differences not only in tracking performance on the MAT but also in levels of perceived workload. With the addition of a measure of workload, we should be able to identify not only individuals with optimal and less than optimal performance, but also distinguish between those who are reporting greater vs. lower levels of workload, even among those who are exhibiting good task performance. We will pay special attention to mental workload, referred to as Mental Demand on the NASA-TLX. In this way, by not merely relying on behavioral task performance, we can better describe the cognitive status of these individuals with HIV infection. And lastly, to better understand possible factors associated with individual differences, various demographic and clinical variables were examined in relation to mental workload (Mental Demand).

## Methods

### Participants

Participants in the present study included 32 adults living with HIV. They were recruited from a larger parent study funded by the National Institute of Mental Health (R01 MH 58552, awarded to Charles Hinkin) that examined cognitive status and medication adherence in adults with HIV. The present study, funded by the National Institute on Aging (R03 AG18549, awarded to David Hardy), took place at the same time and location as the parent study. HIV-positive participants were recruited from an infectious disease clinic at the University of California, Los Angeles, and from local community agencies in the Los Angeles area specializing in services for HIV-positive individuals. All testing and assessments were conducted at the VA Greater Los Angeles Healthcare System. Inclusion/exclusion criteria for participation was: (a) at least 21 years of age, (b) no evidence of any central nervous system opportunistic infection or neoplasm (e.g., progressive multifocal leukoencephalopathy, toxoplasmosis, etc.), (c) no current or history of psychotic spectrum disorder (including bipolar disorder), (d) no history of head injury with loss of consciousness in excess of 30 min, and (e) currently taking highly active antiretroviral treatment. Mean age of participants was 42.47 years (*SD* = 9.86), with 28 males and four females. Mean years of education was 13.33 years (*SD* = 2.79). Participants included African Americans (*n* = 19), White participants (*n* = 8), Asian Americans (*n* = 2), Latino participants (*n* = 1), and Native Americans (*n* = 2). Median CD4 lymphocyte (or white blood cell) count (cells/mm^3^), indicative of immune system functioning in the host, was 287, and median viral load (log 10) was 4.00, with 18 participants meeting diagnostic criteria for AIDS based either on a CD4 count below 200 or the presence of certain opportunistic infections (*pneumocystis carinni* pneumonia, certain cases of candidiasis, cytomegalovirus, etc.).

### Assessment of Multi-Tasking and Workload

For all participants, workload, *via* the NASA Task Load Index, was assessed in the context of single, dual, and tri-task conditions of a tracking task, provided by the Multi-Attribute Task Battery. The MAT is described first and then the NASA-TLX.

#### Multi-Attribute Task Battery

**Figure 1:**
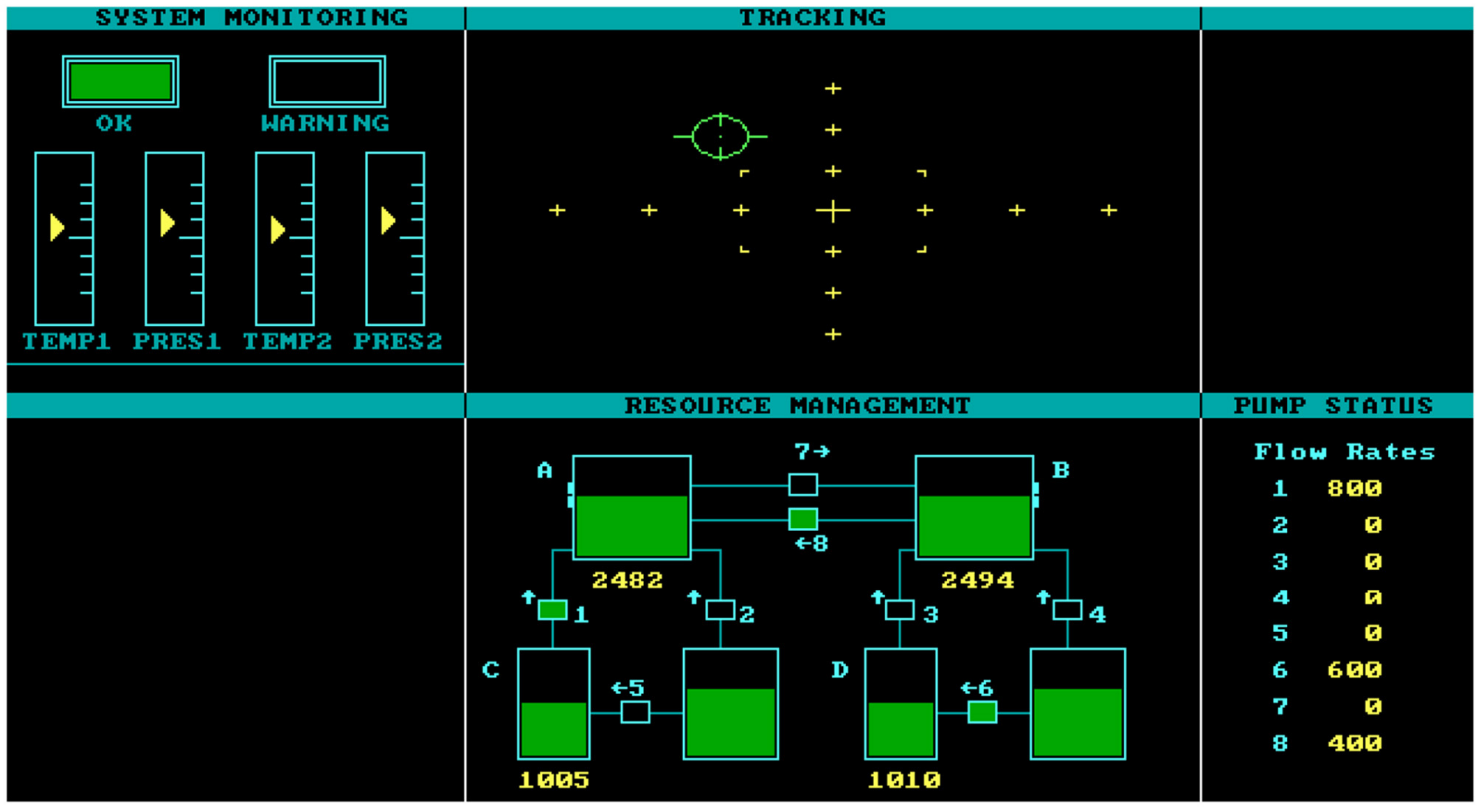
The Multi-Attribute Task.

Originally developed by researchers at Langley Research Center for the National Aeronautics and Space Administration (Comstock and Arnegard, 1992; see also Cegarra et al., 2020; https://matb.larc.nasa.gov), the Multi-Attribute Task (MAT) Battery is a desktop computer simulation of the multi-task environment of the cockpit for laboratory studies of workload and strategic behavior (see Figure 1). Because of the relatively low fidelity of the cockpit environment in the MAT Battery, it is suitable for the assessment of multi-task performance in adults without any flying or piloting experience (e.g., see Hardy et al., 1994; Hardy and Mitrovich, 2008). In addition, because of the flexibility of the MAT, it is an ideal laboratory tool to assess complex performance, performance more complex than the typical neuropsychological test or information processing task. In addition, an assessment of workload (the NASA-TLX), described below, can also be programmed into the MAT Battery framework.

The MAT Battery consists of three component tasks: tracking, system monitoring, and fuel management (see Figure 1). These are described below. Participants initially performed each component task by itself to establish baseline performance levels. Participants then performed two dual-task conditions: (1) tracking and system monitoring and (2) tracking and fuel management. Participants then completed the multi-task condition where all three tasks are performed simultaneously. Participants were instructed to make the tracking task a priority in the dual-task and multi-task conditions. Participants therefore completed six blocked conditions of the MAT Battery. Each block was 10 min long. Total time on the MAT Battery was ~90 min (this includes 60 min actual task time plus time for instructions, practice, workload ratings, and rest breaks between each condition).

In the tracking task (upper-middle window in Figure 1), a green circular target-symbol fluctuated within the window. The participant's task was to keep the target inside the rectangular-shaped inside border at the window center using a joystick. If no control input is applied, the target drifts away from the center toward the edges of the window. Combined root-mean-square errors (RMSEs) are computed for the samples obtained over each one-second period and averaged over a 10-min period to yield a mean RMSE score, with higher scores indicating worse performance.

The system monitoring task (shown in the upper-left window in Figure 1) consisted of four vertical gauges with moving pointers, a green “OK” light, and a red “Warning” light. The gauges are labeled from left to right, TEMP1, PRES1, TEMP2, and PRES2 (representing temperature and pressure status for two engines). When the green “OK” light is on, the pointers fluctuate in each direction within a fixed range around the center of the gauge. When the red “Warning” light is on, the pointer on one of the gauges has gone “off limits” or beyond the fixed range around the center of the gauge (representing a system malfunction). Participants were instructed to respond to the red “Warning” light, to correct the system malfunction, by pressing one of the four keys on the computer keyboard (keys 1, 2, 3, or 4, of the number keys near the top of the keyboard, which were labeled as T1, P1, T2, and P2) that corresponded with the four gauges. Upon a correct participant response, the “off limits” pointer moved immediately back to the center pointer of the gauge and remained there for 1.5 s. Otherwise, the pointer reset itself after 10 s (if no correct response was made) and the green “OK” light returned. Hits, misses, false alarms, and detection errors were recorded, but this data is not presented in the current paper.

For the fuel management task (lower-middle window in Figure 1), the goal is to maintain a specific level of fuel within both of the main tanks (tanks A and B). The display for this task consists of six rectangular regions that represent fuel tanks. Along the fuel lines that interconnect these tanks are fuel pumps capable of transferring fuel in one direction and at a specified flow rate. Therefore, to maintain the task objective, participants must transfer fuel from the supply tanks using one or more of the eight fuel pumps. Pumps are activated by a corresponding key press (numbers 1 through 8 on the keyboard number pad). A global measure of task performance is obtained by computing the mean root-mean-square error (RMSE) in the fuel level of tanks A and B (deviation from the required level of 2,500 gallons). This data is not presented in the current paper.

#### NASA Task Load Index

**Figure 2:**
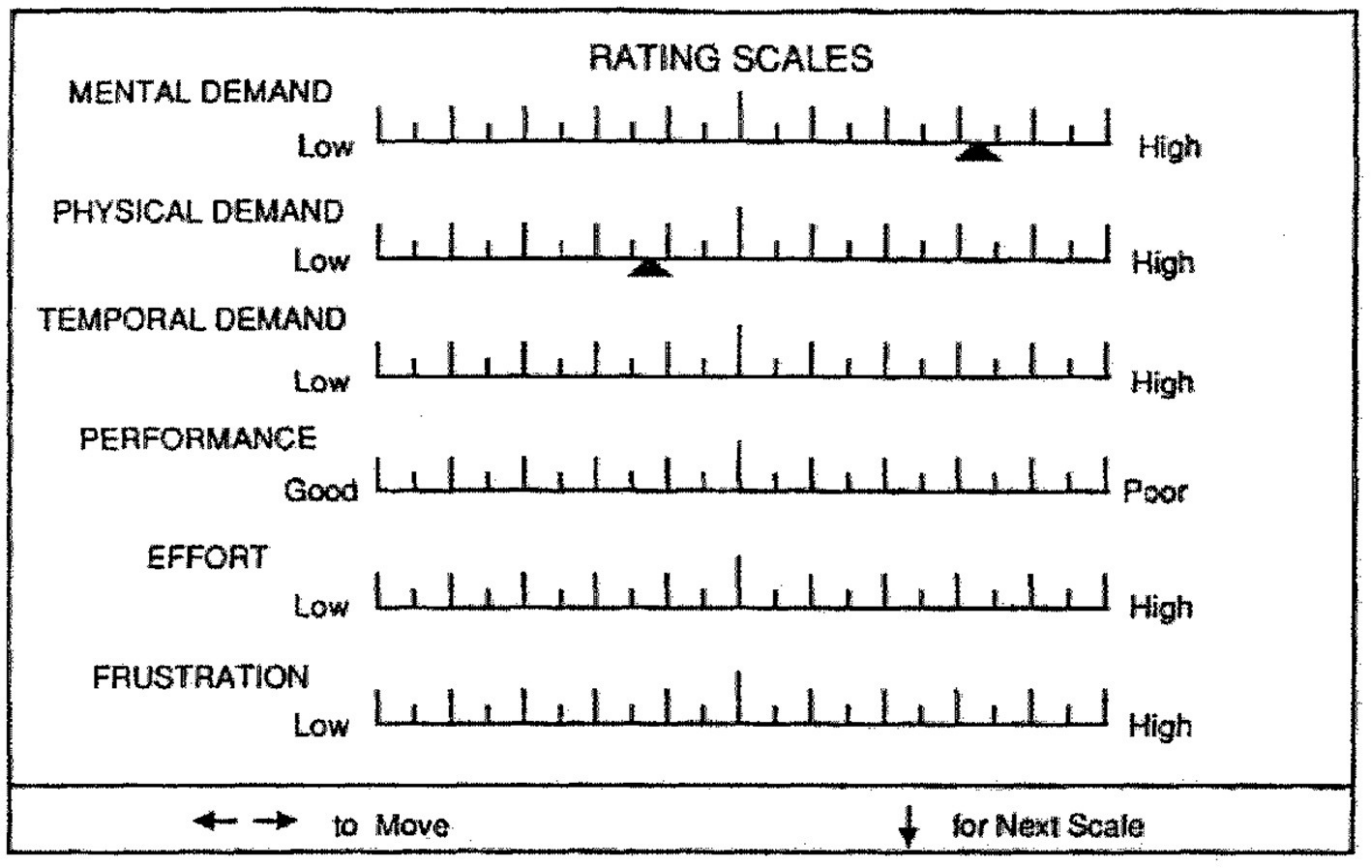
The NASA Task Load Index (NASA-TLX).

Workload was assessed with the NASA Task Load Index (NASA-TLX), a commonly used self-report measure of workload (Hart and Staveland, 1988; Hart, 2006; see www.nasatlx.com). The NASA-TLX includes six subscales: Mental Demand, Physical Demand, Temporal Demand, Effort, Frustration, and Performance. Participants rated their perception of exertion on these subscales from “Low” to “High” except for the Performance subscale which ranged from “Good” to “Poor” (see Figure 2). In the computerized version used in the present study, the marker for each subscale was manipulated with the computer mouse with each subscale score ranging from 0 to 100 (numerical scores were not visible to the participant). Overall workload was also calculated as the average of the six subscales. The NASA-TLX is often used in conjunction with simulators or tasks that emulate real-world scenarios such as operating an aircraft, car, tank, and the like, to assess dimensions of workload in these situations. Here, participants completed the NASA-TLX at the end of each block of trials on the Multi-Attribute Task Battery (described above). A description of each workload subscale was provided to each participant (see Table 1). Ultimately, three sets of NASA-TLX scores were analyzed in the present report: (1) in a single task condition where the MAT tracking task was performed alone, (2) in a dual-task condition where tracking was performed along with the system monitoring task, and (3) in a tri-task condition, which involved tracking, system monitoring, and fuel management.

**Table 1:** NASA-Task Load Index rating subscale descriptions.

**Subscale title**	**Description**
Mental Demand	How much mental and perceptual activity was required (e.g., thinking, deciding, calculating, remembering, looking, searching, etc.)? Was the task easy or demanding, simple or complex, exacting, or forgiving?
Physical Demand	How much physical activity was required (e.g., pushing, pulling, turning, controlling, activating, etc.)? Was the task easy or demanding, slow or brisk, slack or strenuous, restful, or laborious?
Temporal Demand	How much time pressure did you feel due to the rate or pace at which the tasks or task elements occurred? Was the pace slow and leisurely or rapid and frantic?
Performance	How successful do you think you were in accomplishing the goals of the task set by the experimenter (or yourself)? How satisfied were you with your performance in accomplishing these goals?
Effort	How hard did you have to work (mentally and physically) to accomplish your level of performance?
Frustration	How insecure, discouraged, irritated, stressed and annoyed versus secure, gratified, content, relaxed, and complacent did you feel during the task?

## Results

As mentioned above, tracking performance on the MAT Battery was measured as the average deviation (RMSE) from the designated area where the tracking symbol should be kept within *via* the control of a joystick. Tracking performance was examined with a within-subjects one-factor analysis of variance (ANOVA), with tracking task condition (single, dual, and tri-task) as the within-subjects factor. Performance significantly differed among the three task conditions, *F*_(2, 62)_ = 74.96, *p* < 0.001, η^2^ = 0.71, with tracking performance getting progressively worse with task condition difficulty (see Table 2). Similar ANOVAs were conducted on the NASA-TLX measures, Overall Workload and the six subscales. As can be seen in Table 2, there was a significant difference in NASA-TLX ratings (as a function of tracking task condition) for all measures except the Performance subscale. Effect size, calculated as partial eta square, ranged in value from 0.00 to 0.35 for the NASA-TLX ratings, with the largest values for Overall Workload (η^2^ = 0.35), Mental Demand (η^2^ = 0.33), and Physical Demand (η^2^ = 0.32).

**Table 2:** Means (and standard deviations) for tracking performance on the Multi-Attribute Task (MAT) and workload ratings on the NASA-TLX in adults with HIV.

	**Tracking task condition**					
	**Single (A)**	**Dual (B)**	**Tri (C)**	***P***	**η^2^**	***Post-hoc***
Tracking Perf. (RMSE)	21.56 (13.96)	31.77 (19.01)	49.52 (27.83)	<0.001	0.71	A < B < C
NASA-TLX						
Overall Workload	54.30 (17.48)	60.57 (18.83)	65.90 (17.32)	<0.001	0.35	A < B < C
Mental Demand	56.25 (24.36)	64.44 (22.52)	71.63 (22.14)	<0.001	0.33	A < B < C
Physical Demand	45.63 (21.94)	60.22 (23.30)	63.72 (23.75)	<0.001	0.32	A < B < C
Temporal Demand	48.00 (24.35)	54.66 (23.65)	58.31 (27.42)	<0.033	0.10	–
Effort	57.06 (22.32)	64.03 (22.02)	74.34 (25.03)	<0.001	0.20	A < B & C
Frustration	43.78 (22.24)	53.09 (27.14)	58.47 (28.80)	<0.004	0.17	A < C
Performance	45.84 (22.03)	46.88 (22.59)	46.56 (20.49)	<0.949	0.00	–

*A pairwise post hoc comparison is significant (at a Bonferonni adjusted 0.05) as indicated*.

**Figure 3:**
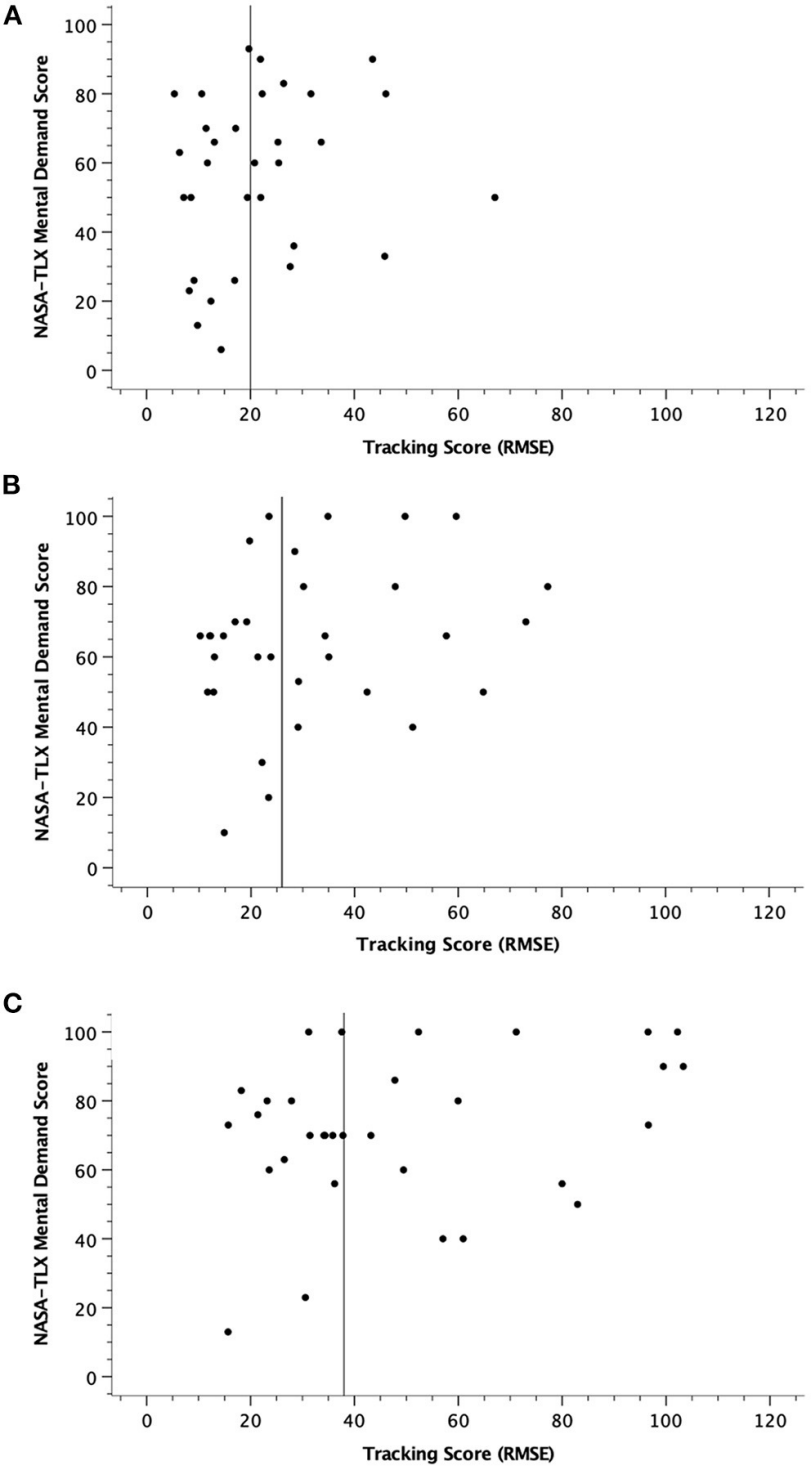
Scatterplots of NASA-TLX Mental Demand scores and MAT tracking scores in single task **(A)**, dual-task **(B)**, and tri-task **(C)** tracking conditions.

Individual differences in workload were examined visually or graphically with scatterplots. More specifically, and with an intention to keep the paper more focused and of a reasonable length, of the seven workload measures of the NASA-TLX, we chose Mental Demand as the outcome of most interest. Three scatterplots were constructed (see Figure 3), with Mental Demand scores plotted on the Y-axis and tracking performance (RMSE) plotted on the X-axis for the single, dual, and tri-task conditions. A reference line was inserted at the median tracking score for each scatterplot. As is evident, there was much variability in tracking performance and Mental Demand ratings. One point of interest in these scatterplots is the evidence of notably high Mental Demand ratings, not just in the those with poorer tracking performance (higher RMSE scores indicate worse performance), but also in some individuals with better performance (i.e., those with lower RMSE scores). This is especially the case in the tri-task condition. To examine the association between tracking performance (RMSE) and Mental Demand rating, a concomitant Pearson correlation analysis was conducted for each scatterplot, with coefficients of *r* = 0.19 (*p* = 0.312) for the single tracking condition, *r* = 0.25 (*p* = 0.175) for dual, and *r* = 0.30 (*p* = 0.096) for the tri-task condition.

To examine variables with a potential impact on workload, several variables were examined in relation to Mental Demand scores *via* correlation analyses. These are shown in Table 3. As can be seen, age was significantly associated with Mental Demand ratings in all three MAT tracking conditions. Somewhat weaker associations with Mental Demand were found with AIDS status (yes or no) and also presence (or not) of HIV-related opportunistic infections.

**Table 3:** Correlations between demographic, psychological, and medical variables with Mental Demand ratings on the NASA-TLX in adults with HIV.

	**Mental Demand/tracking task condition**		
	**Single**	**Dual**	**Tri**
Age	0.41 (0.022)	0.48 (0.006)	0.45 (0.009)
Education	0.09 (0.626)	0.16 (0.379)	0.06 (0.766)
Gender	0.07 (0.716)	0.02 (0.922)	0.11 (0.563)
BDI-II	0.19 (0.310)	0.13 (0.479)	0.04 (0.823)
CD4 count (cells/mm^3^)	0.09 (0.643)	0.11 (0.569)	0.07 (0.704)
Viral load (log 10)	0.07 (0.718)	0.07 (0.724)	0.06 (0.760)
AIDS diagnosis	0.20 (0.273)	0.26 (0.152)	0.34 (0.066)

*Correlation coefficients are presented with p-values inside parentheses*.

## Discussion

With all the task measures and subscales, and the different kinds and combinations of analyses that are possible, assessments like the MAT and the NASA-TLX generate a great deal of data. A preliminary set of analyses are presented here, in the exploration of several issues as it relates to adults with HIV, and to neuropsychology in general.

As an assessment instrument of multi-tasking ability and the requisite cognitive processes involved (such as executive functions, divided attention, and other aspects of attention, etc.), the MAT showed large differences in tracking performance among the single, dual, and tri-task conditions. Indeed, an η^2^ of 0.71 is a considerable effect size. This finding reflects the efficacy of the MAT as a useful research tool. As far as we know, because this is an experimental task, there are no normative data sets available with which to gauge the performance of our HIV-positive group. In addition, without a proper control group, it is impossible to directly assess the absolute level of MAT performance here. In other words, how good or bad did our participants perform on the tracking task relative to how others perform? Although admittedly an imperfect comparison group, we do have data on the performance of 18 college students on a similar version of the MAT Battery (Hardy and Mitrovich, 2008). In this group, tracking performance in single (*M* = 10.41, *SD* = 2.33), dual (*M* = 14.24, *SD* = 2.98), and tri-task (*M* = 20.06, *SD* = 4.89) conditions was considerably better than in our current HIV-positive group. This difference in performance is not surprising considering the college group was younger and without any serious medical issues. In addition to a basic group difference, there were other noticeable differences in performance. For instance, in the college sample, tracking task performance accuracy (RMSE) declined by 37 and 93% in the dual and tri-task conditions, respectively, compared to single-task tracking performance. In the current HIV-positive group, tracking task performance decrements were at 47 and 130% for dual and tri-task conditions. So again, although these larger decrements in the HIV-positive group are not surprising, they do provide a sense of the degree of challenge these individuals experienced on the MAT.

These multi-tasking costs in tracking performance on the MAT are considerably larger compared to dual-task costs we have observed in HIV-positive adults on more traditional reaction time tasks, where average response time was delayed around 20% in dual-task versus single-task conditions (Hinkin et al., 2000). Of course, the nature of the MAT Battery, where performance is continuous and more similar to the real-world operation of an aircraft and other types of vehicles, is markedly different compared to the trial-by-trial context of most typical laboratory tests of cognition. That is what makes the MAT an attractive assessment tool in human factors and neuroergonomics research. Its usefulness in neuropsychology probably depends on the goal of the assessment. For use as a standard clinical assessment, the MAT Battery is probably too long, too time intensive. And no test norms are currently available. In addition, a computer is obviously necessary, something that is still not commonplace in all clinical settings (e.g., see Kessels, 2019). On the other hand, as a research tool in neuropsychology, the MAT Battery has many attractive features. For the assessment of multi-tasking ability, attention, continuous tracking, and other related cognitive abilities, the MAT provides a powerful assessment. It has flexibility to match the particular needs of the researcher (the program code can be modified), and a greater verisimilitude to real-life behavior than most neuropsychological tests. And lastly, as government software in the public domain, the MAT Battery is free (see Cegarra et al., 2020) which could facilitate its adoption in diverse settings beset by funding limitations.

As the MAT tracking task became more difficult in single, dual, and tri-task conditions, as evidenced by a progressive decline in performance, self-report of workload went up. This was evident in Overall Workload as well as all subscales except for Performance. Effect size, as measured as eta squared, was largest in Overall Workload (η^2^ = 0.35), followed closely by Mental Demand (η^2^ = 0.33). These findings were expected and make sense. Capturing all of the assessed elements of workload, Overall Workload would be expected to be the most sensitive measure.

As with the MAT Battery (and most experimental laboratory tasks), test norms on the NASA-TLX are not available. Even if there were, these would need to be specific to our particular configuration of the MAT. However, some general and limited comparisons are possible. For instance, in an effort to improve interpretation of NASA-TLX scores, a meta-analysis was conducted on the existing studies at the time that used the NASA-TLX (Grier, 2015). This resulted in over a thousand global workload scores over 200 published studies. These were categorized by type of task that was used in conjunction with the NASA-TLX, with some task categories being relevant to the present study. For instance, for “Tracking Tasks”, median global or overall workload was 51.00. This is close to but under the mean Overall Workload rating in single-task tracking (*M* = 54.30) in the present study, and further away from the dual-task (*M* = 60.57) and tri-task (*M* = 65.90) workload ratings in our HIV-positive adults. Not to make too much of these imperfect comparisons, but it should be noted that although Grier (2015) did not differentiate tasks by multitasking status (i.e., was the task completed alone or concomitantly with other tasks?), she did note that “Many of the publications examined workload in multi-tasking situations” (p. 1,730). So her values, general as they are, suggest that workload ratings in the HIV-positive group were perhaps somewhat higher than what would normally be expected. This is not a strong argument we are making, but along this same line of reasoning, when looking at Grier's global workload rating scores across all studies, a score of 53.97 is at the 60th percentile, a score of 60 is at the 75th percentile, 62 is at the 80th percentile, with 68 at the 90th percentile. Thus, the Overall Workload scores in the present study are all in the top half of this broad and general distribution of workload scores, suggesting that, in general, the HIV-positive group in our study were experiencing high levels of workload while performing the MAT. That said, we are not interpreting these percentiles as “cut-scores” (as they say in neuropsychology). Likewise, in the Grier paper, she asserted that her percentiles did not necessarily indicate demarcation points for a “redline” (as they say in human factors and neuroergonomics).

The Mental Demand subscale of the NASA-TLX was of particular interest to us. Ratings on this subscale were similar to Overall Workload, with a similar and large effect size. As anticipated, Mental Demand rating on the MAT tracking task increased incrementally from single, dual, and tri-task conditions. Large individual differences were also evident in the relationship between Mental Demand ratings and tracking performance. As can be seen in Figure 3, even among the better performers on the MAT tracking task, say for instance, those with RMSE scores below the median (lower scores are better scores), there is a large range of Mental Demand scores, with some reporting low levels while others are reporting fairly high levels of Mental Demand. This is especially the case in the tri-task condition. This is admittedly a simple qualitative analysis, although the non-significant correlation analyses between tracking and Mental Demand ratings support the notion of large individual differences across both variables. But the question can still be asked, among the HIV-positive participants with optimal tracking performance on the MAT, is the cognitive status of those reporting high levels of Mental Demand the same as those reporting lower levels? We suggest that the cognitive status in these two scenarios is not necessarily the same.

Because workload is the interaction between demands of the task at hand as well as the capacities (or cognitive resources) of the individual, there are a variety of factors that could potentially impact it. Continuing our focus on the Mental Demand subscale of the NASA-TLX, we examined the association between a variety of such factors and Mental Demand ratings in the three tracking task conditions (see Table 3). The largest correlation coefficients were found with participant age. These were positive correlations, with Mental Demand scores getting larger with older age. Calculating simple coefficients of determination, age accounted for 16.81, 23.04, and 20.25% of Mental Demand ratings variance in single, dual, and tri-task conditions respectively. A diagnosis of AIDS (based on the presence of opportunistic infections or a CD4 count lower than 200 cells/mm^3^) showed suggestive (but statistically insignificant) correlations, especially in the tri-task condition (*p* = 0.066). With a suppressed immune system, and because history of opportunistic infection was reported in 21 individuals, this suggests that the medical state of the individual could potentially negatively impact workload, although not all of these individuals were necessarily experiencing an opportunistic infection during testing. Such a finding is compatible with a recent conceptualization of mental workload, where workload is partially determined by the general activation or level of energetic arousal (as part of what they refer to as *germane load*) of the individual (Galy et al., 2018). In the present study, then, the HIV-positive adults with an AIDS diagnosis (including many with a history of opportunistic infection) would be considered to perhaps have a reduced level of energetic arousal, resulting in an increase in Mental Demand. This is admittedly a *post hoc* assessment of these individuals, and warrants further investigation in the future.

Other NASA-TLX subscales will be briefly mentioned at this point. For instance, Effort showed a similar pattern to Overall Workload and Mental Demand. Physical Demand also showed this pattern. Although the relationship between mental workload and physical workload is a complex and intriguing one (Young et al., 2015; Galy et al., 2018), the increased ratings of Physical Demand in the present study make sense on the face of it. All three tasks on the MAT Battery involved motor responses with the hand. Performing the tracking task alone required a hand on the joystick to constantly correct the position of the moving target marker on the screen. In the dual and tri-task conditions, the system monitoring and resource management tasks required not only visual scanning and other cognitive processes but also frequent button presses, thus requiring the use of both hands simultaneously. Thus, it makes sense that the largest difference in Physical Demand rating is between the single task condition (where only one hand was used) and the dual and tri-task conditions (where each required both hands), where the difference between the latter two conditions was smaller.

For the Performance subscale, participants rated how they thought they performed on the tracking task. Interestingly, this is the one NASA-TLX subscale that did not show significant differences across tracking conditions. In fact, Performance ratings were almost identical (*p* = 0.949), despite actual task performance declining in a stepwise manner in single, dual, and tri-task conditions. This finding is somewhat puzzling because the Performance subscale is sensitive to task difficulty. For instance, in a study looking at the performance of a problem-solving test with three levels of difficulty (the Tower of Hanoi test with three, four, and five disks), NASA-TLX Performance scores declined from 76.03 to 64.97 to 48.07 (*p* < 0.001) (Hardy and Wright, 2018). The effect size was a substantial η^2^ of 0.45, and this was found in young healthy college students. Participants in the present study were not college students but adults with HIV (with some considerably older than college students), and an intriguing finding with this population might be relevant here. Several studies have shown subgroups within HIV-positive adults that show poor insight with regards to their own cognitive status (van Gorp et al., 1991; Wilkins et al., 1991; Hinkin et al., 1996; Thames et al., 2011). Most relevant to us are those who seem unaware of their own cognitive impairment. For instance, in one study, 26% of HIV-positive adults self-reported having no cognitive impairment when actually showing deficits on a variety of memory measures (Hinkin et al., 1996; see also Thames et al., 2011 for a similar finding). In the present study, although it is unclear if the HIV-positive group is impaired on MAT tracking performance (although there are some suggestions based on imperfect comparison data that this might be the case), that their self-assessment of performance did not change with task difficulty, even when their actual performance did change (as did their self-ratings of workload), is reminiscent of these previous findings of reduced insight or metacognition. So, did they actually think they performed the same across tracking task conditions? Or did they realize the task was more difficult and make a “mental correction” to conclude that their performance level remained satisfactory even as their actual performance declined as the task became more difficult (e.g., “given how hard that last condition was I think I did just fine”). A closer and prospective examination of this issue is warranted, to determine if and to what degree an anosognosia is present and how this might be related to various aspects of workload, performance, and other potentially relevant variables (e.g., mood, medical status including the diagnosis of AIDS, etc.).

In conclusion, although preliminary in nature due to the lack of a proper control group, results from the present study illustrate the presence of large individual differences in mental workload, which to a certain degree, were independent of tracking performance. We argue that a difference in workload, even with comparable levels of behavioral performance, might be indicative of a difference in cognitive status. In addition, variables such as age and an AIDS diagnosis related to mental workload were also identified. Although no association with gender was evident, the present group of HIV-positive adults was heavily skewed toward males, so this issue remains for further examination in the future. Another issue to note is the method of workload assessment in the present study. Although the NASA-TLX is a relatively simple subjective assessment of workload, there are many objective measures of workload, with behavioral and physiological options (for reviews, see Vidulich and Tsang, 2012; Ranchet et al., 2017), that can be considered for future neuropsychological studies. A potential deficit in metacognition also surfaced, requiring a closer examination in a future study. Levels of workload might also prove to be an important factor in overall health status in clinical populations, possibly relating to real-world outcomes such as work and school performance, the enjoyment of hobbies, effective functioning at home, and other activities of daily living, another topic worthy of future examination. Ultimately, because large individual differences are a hallmark of the neurological and cognitive sequelae of adults with HIV, and in many other clinical syndromes, the assessment of workload and factors related to workload could be a valuable addition in neuropsychology.

## Data Availability Statement

The original contributions presented in the study are included in the article/supplementary files, further inquiries can be directed to the corresponding author/s.

## Ethics Statement

The studies involving human participants were reviewed and approved by the Institutional Review Board (IRB) of UCLA and the VA Greater Los Angeles Healthcare System. The patients/participants provided their written informed consent to participate in this study.

## Author Contributions

DH contributed to conceptual development, task programming, statistical analyses, results interpretation, and writing of the manuscript. CH contributed to conceptual development, results interpretation, and writing of the manuscript. Both authors contributed to the article and approved the submitted version.

## Funding

National Institute of Mental Health R01 MH 58552/funds provided for CH parent study (test materials, subject payment, staff payment, etc.) and National Institute on Aging R03 AG 18549/funds provided specifically for DH current study (test materials, subject payment, partial salary for DH, etc.).

## Conflict of Interest

The authors declare that the research was conducted in the absence of any commercial or financial relationships that could be construed as a potential conflict of interest.

## Publisher's Note

All claims expressed in this article are solely those of the authors and do not necessarily represent those of their affiliated organizations, or those of the publisher, the editors and the reviewers. Any product that may be evaluated in this article, or claim that may be made by its manufacturer, is not guaranteed or endorsed by the publisher.
